# New York State Board of Veterinary Examiners

**Published:** 1895-10

**Authors:** W. H. Kelly

**Affiliations:** Secretary


### NEW YORK STATE BOARD OF VETERINARY EXAMINERS.

SECOND meeting of the Board of Veterinary Examiners, regents of the State of New York, was held in New York, Friday evening, September 6th. The entire board present: Professor Law, President; Dr. W. H. Kelly, Secretary; Drs. Hinkley, Huidekoper, and Morris. After consideration of the colleges to be recognized by the Board of Regents of New York State, it was unanimously decided that the following are properly equipped and give the necessary three-years' instruction by a full staff of veterinary instructors:

The New York College of Veterinary Surgeons, New York City.

The American Veterinary College, New York City.

The Veterinary Department of the University of Pennsylvania, Philadelphia.

The School of Veterinary Medicine, Harvard College, Boston, Mass.

The McKillip Veterinary College, Chicago, Ill.

The Veterinary College at Ames, Iowa.

The School of Comparative Medicine of McGill University, Montreal, Canada.

All veterinary colleges under government control in the continental countries of Europe.

All veterinary colleges in Great Britain and Ireland whose students are licensed by the Royal College of Veterinary Surgeons.

The board then proceed with the selection of questions for the first examinations, September 24th to 27th, at New York, Albany, Syracuse, and Buffalo, which were placed under seal and forwarded to the Board of Regents.

W. H. KELLY,
Secretary.

